# Ambulatory ECG monitoring for syncope and collapse in United States, Europe, and Japan: The patients’ viewpoint

**DOI:** 10.1002/joa3.12560

**Published:** 2021-05-24

**Authors:** Meltem Altinsoy, Richard Sutton, Ritsuko Kohno, Scott Sakaguchi, Robin K. Mears, David G. Benditt

**Affiliations:** ^1^ Cardiac Arrhythmia Center Cardiovascular Division University of Minnesota Medical School Minneapolis MN USA; ^2^ Cardiology Residency Turgut Ozal University School of Medicine Ankara Turkey; ^3^ Cardiology Department National Heart and Lung Institute Imperial College Hammersmith Hospital London UK; ^4^ Medtronic Inc. Minneapolis MN USA

**Keywords:** ambulatory ECG monitoring, insertable cardiac monitors, syncope

## Abstract

**Background:**

Practice guidelines provide clinicians direction for the selection of ambulatory ECG (AECG) monitors in the evaluation of syncope/collapse. However, whether patients’ understand differences among AECG systems is unknown.

**Methods and Results:**

A survey was conducted of USA (n = 99), United Kingdom (UK)/Germany (D) (n = 75) and Japan (n = 40) syncope/collapse patients who underwent diagnostic AECG monitoring. Responses were quantitated using a Likert‐like 7‐point scale (mean ± SD) or percent of patients indicating a Top 2 box (T2B) for a particular AECG attribute. Patient ages and diagnosed etiologies of syncope/collapse were similar across geographies. Patients were queried on AECG attributes including the ability to detect arrhythmic/cardiac causes of collapse, instructions received, ease of use, and cost. Patient perception of the diagnostic capabilities and ease of use did not differ significantly among the AECG technologies; however, USA patients had a more favorable overall view of ICM/ILRs (T2B: 42.4%) than did UK/D (T2B: 28%) or Japan (T2B: 17.5%) patients. Similarly, US patient rankings for education received regarding device choice and operation tended to be higher than UK/D or Japan patients; nevertheless, at their best, the Likert scores were low (approximately 4.7‐6.0) suggesting need for education improvement. Finally, both US and UK/D patients were similarly concerned with ICM costs (T2B, 31% vs 20% for Japan).

**Conclusions:**

Patients across several geographies have a similar but imperfect understanding of AECG technologies. Given more detailed education the patient is likely to be a more effective partner with the clinician in establishing a potential symptom‐arrhythmia correlation.

## Graphical abstract

We surveyed syncope/collapse patients from three geographical regions (United States, United Kingdom/Germany and Japan) who underwent AECG monitoring. Patients across several geographies have similar, albeit imperfect, understanding of AECG technologies, and warrant more complete education regarding AECG devices selected for their care.

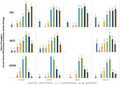

## 1 INTRODUCTION

Establishing the cause of syncope and collapse remains a challenge.^1^, ^2^ In this regard, practice guidelines and consensus statements provide practitioners with appropriate diagnostic direction.^3^, ^4^, ^5^, ^6^, ^7^ In particular, it is agreed that the first steps should comprise a medical history detailing overall health, past medical history, and current symptoms; thereafter, physical examination and selected laboratory tests based on the clinical history (eg, ECG, echocardiogram) are appropriate. However, if the diagnosis continues to be unclear and an arrhythmic cause remains a concern, practice guidelines provide strategies for appropriate use of shorter‐ and longer‐term ambulatory ECG (AECG) monitoring devices in an attempt to document subsequent symptomatic episodes or potentially causal arrhythmias.^3^, ^4^, ^5^, ^6^, ^7^ In brief, while the guidelines cannot specify specific values for sensitivity and specificity of monitoring device types since clinical circumstances vary widely, they do teach that for maximum diagnostic sensitivity, the monitoring duration should be selected based on the expected event frequency; specifically, low event frequencies require longer monitoring technologies. Thus, external wearable AECG monitors (ECM) are recommended for those patients in whom spontaneous symptoms are relatively frequent (eg, daily to monthly); for longer inter‐event intervals (eg, >30 days apart), insertable cardiac monitors (ICM) are advocated.

Previous reports indicate that the majority of physicians recommend AECG technology appropriately according to guidelines.^8^, ^9^ However, the extent to which patients understand differences among the AECG options selected for their care, and consequently the basis for practitioner recommendations is unclear; greater patient understanding of prescribed technology may be expected to lead to greater acceptance and enhanced compliance with a provider‐proposed AECG monitoring strategy. In particular, informed patients may be expected to be more attentive to and more successful with capturing ECG findings during symptomatic events (ie, potential symptom‐arrhythmia correlations).

The survey reported here was designed to use ‘real world’ data,^10^, ^11^ with three overall objectives. First was to assess, among syncope/collapse patients residing in three differing geographies (USA, United Kingdom and Germany [UK/D], and Japan) who have undergone AECG monitoring, their understanding of the range of AECG technologies and the relative utility of various wearable and insertable diagnostic AECG monitoring systems. Second, we aimed to assess the relative frequency with which various AECG technologies were employed in the geographies evaluated. Finally, we examined patients’ perspectives regarding factors affecting patient acceptance of and compliance with the various AECG technologies.

## 2 METHODS

This report comprises findings obtained from a survey undertaken of patients residing in the USA (n = 100), UK/D (n = 75), and Japan (n = 40) in whom AECG monitoring had been experienced as part of an evaluation into the cause of their syncope/collapse. Patients were selected from those presenting with syncope/collapse to Emergency Departments, Cardiology, or Neurology clinics. Potential candidates were identified with the aid of attending physicians, and patients were subsequently qualified by direct communication by an independent polling agency (see below) as having had syncope/collapse and having experienced ambulatory monitoring. The population size was limited by the complexity of data collection across diverse clinics and geographies. Except for mobile cardiac outpatient telemetry (MCOT) which was not widely applied in Europe at the time, the remaining technologies were widely used. The survey was conducted by an independent polling agency (ZS Associates).

In the absence of prior studies offering an estimated difference in patient responses regarding cardiac monitoring in different geographies, the required size of the patient population to observe statistically significant differences was based on estimates guided by the polling agency experience. We assumed that USA and UK/D might be similar in many respects but that their diversity of the population was greater than that of Japan. Consequently, the patient number of patients surveyed was approximately twice as many in USA and UK/D than in Japan.

Respondents completed a quantitative survey instrument online. As part of the survey, patients were queried regarding their understanding of the attributes of the various available AECG technologies. Simple descriptive materials were used to refresh the patient recollection of the various AECG modalities and terminology.

Surveyed patients individually provided informed consent for use of their data. Each participant was informed that they would remain anonymous and that all information gathered would be confidential. Investigational approval was obtained from the appropriate ethics committee at each data collection site. Responding patient's each received an honorarium of US$50. Medtronic Inc sponsored the survey and a sponsor co‐author representative (RKM) assisted with technical aspects of the survey and reviewed the manuscript. However, the sponsor was not involved with data collection or the interpretation of the findings provided in this manuscript.

Non‐dichotomous responses were graded using a Likert‐like 7‐point scale (0 = I don't know, 1 = Not important, 4 = Somewhat important, 7 = Very important with grades 5 and 6 offering the responder additional discrimination).^10^ A Top 2 Box (T2B, combined grades 6 and 7) score was used to provide a way of summarizing the positive responses from the Likert scale survey question.

### 2.1 Statistical analysis

Whether the distribution of discrete variables was normal or not was determined by Kolmogorov‐Smirnov test. Levene test was used for the evaluation of homogeneity of variances. The descriptive statistics for discrete and ordinal variables were shown as mean ± SD (min‐max). The number of cases and percentages were expressed as categorical data.

Differences in mean ages of patients by country were compared by one‐way ANOVA, otherwise, the Kruskal‐Wallis test was applied for the comparisons of variables in which parametrical test assumptions were not met. When the p‐values from Kruskal‐Wallis test statistics were statistically significant, the Dunn‐Bonferroni multiple comparison test was used to know which group differs from which others. Categorical data were analyzed by Pearson's chi‐square test.

Data analysis was performed using IBM SPSS Statistics version 17.0 software (IBM Corporation). Whether differences in assessment scores related to AECG monitoring with various devices were statistically significant was evaluated by Friedman test, otherwise, Cochran's Q test was applied for comparisons of ratios of awareness of cardiac monitoring devices. When the p‐values from Friedman or Cochran's Q test statistics were statistically significant, the Bonferroni Adjusted Wilcoxon Sign Rank or McNemar tests were used to know which device assessment differs from which others. A *P* < .05 was considered statistically significant.

## 3 RESULTS

### 3.1 Respondent characteristics

The survey comprised 215 patients from three different geographical regions (USA, n = 100; UK/D, n = 75; and Japan, n = 40) who underwent AECG monitoring for syncope/collapse. Data was incomplete in one USA externally monitored (ECM) patient whose data was excluded, resulting in a total of 99 USA patients. Among subjects who were originally approached for inclusion response rates were similar in USA and UK/D (approx. 14%), and in Japan (approx.12%). This response rate is consistent with what is expected for comparable Web‐based surveys.

Demographic and clinical diagnostic characteristics of the respondents are presented in Table 1. Other than the Japanese population which had a greater male proportion than the other 2 geographies (*P* < .001 vs USA; *P* < .05 vs UK/D), the patient population ages (mean ± SD; USA 34.7 ± 12.0 years; UK/Germany, 33.5 ± 11.5 years; Japan, 37.6 ± 13.8 years) and presumed etiologies of syncope were similar across geographies. In some patients, multiple causes of syncope were determined to have more than one cause of syncope (eg, orthostatic hypotension and vasovagal syncope) resulting in diagnostic totals >100% in Table 1. Further, patients in all 3 regions sought initial medical evaluation to ascertain the cause of symptoms after fewer than 2 syncope/collapse events (USA, 1.8 ± 5.2; UK/D, 1.2 ± 2.6; Japan 1.5 ± 0.7: USA vs UK/D, *P* = NS; UK/D vs Japan *P* < .046, USA vs Japan, *P* = NS) indicating the importance that they assigned to their symptoms.

**TABLE 1:** Patient characteristics

	USA (n = 99)	UK/Germany (n = 75)	Japan (n = 40)	*P*‐value
Age (y)	34.7 ± 12.0	33.5 ± 11.5	37.6 ± 13.8	.216^†^
Gender				
Female	60 (60.6%)^a^	40 (53.3%)^b^	9(22.5%)^a^, ^b^	
Male	39 (39.4%)^a^	35 (46.7%)^b^	31(77.5%)^a^, ^b^	<.001^‡^
Duration since most recent syncope/collapse (wk)	10.3 ± 12.9	10.7 ± 12.3	10.8 ± 12.6	.305^†^
Presumed etiology of syncope/collapse (patients may have been assigned more than one diagnosis)				
Cardiac	47 (47.5%)	35 (46.7%)	19 (47.5%)	.994^‡^
Neurally‐mediated	54 (54.5%)	40 (53.3%)	23 (57.5%)	.912^‡^
Orthostatic	59 (59.6%)	36 (48.0%)	19 (47.5%)	.227^‡^
Neurological	41 (41.4%)	24 (32.0%)	17 (42.5%)	.375^‡^
Number of episodes before seeking care	1.8 ± 5.2	1.2 ± 2.6^b^	1.5 ± 0.7^b^	**.046** ^†^
Number of events causing injuries	1.4 ± 1.4	1.7 ± 3.0	1.0 ± 1.7	.051^†^

^a^ USA vs Japan (*P* < .001).

^b^ UK/Germany vs Japan (*P* < .05).

^†^ One‐Way ANOVA.

^‡^ Pearson's Chi‐square test.

### 3.2 Respondent understanding of AECG technologies

Based on the type of devices prescribed for and personally experienced by respondents, the various AECG technologies did not differ significantly across the three geographies (Table 2). Not surprisingly, Holter monitors were the most widely used and ICM/ILR tended to be the least used AECG devices. Nonetheless, although not all technologies were used in all patients, patient familiarity with the various technologies was similar across geographies with the exception of MCOT which was not widely available in Europe at the time of the survey (Table 2).

**TABLE 2:** Patient knowledge of specific AECG technology

	USA (n = 99)	UK/D (n = 75)	Japan (n = 40)	*P*‐value^†^
Devices personally used (% patients)				
Holter monitor	78 (78.8%)^a,b,c^	52 (69.3%)^c^	35 (87.5%)^a,c^	.075
Event monitor	43 (43.4%)^a^	37 (49.3%)^d^	21 (52.5%)^a,d^	.562
Mobile cardiac outpatient telemetry	38 (38.4%)^b^	N/A	N/A	—
Insertable cardiac monitor/implantable loop recorder	30 (30.3%)^c^	19 (25.3%)^c,d^	10 (25.0%)^c,d^	.708
*P*‐value^¶^, ^‡^	<0.001	<0.001	<0.001	
Aware of AECG technology (% patients)				
Holter monitor	77 (77.8%)	52 (69.3%)	29 (72.5%)	.445
Event monitor	66 (66.7%)	49 (65.3%)	32 (80.0%)	.228
Mobile cardiac outpatient telemetry	66 (66.7%)	N/A	N/A	—
Insertable cardiac monitor/implantable loop recorder	57 (57.6%)	42 (56.0%)	24 (60.0%)	.918
*P*‐value^¶^, ^‡^	0.019	0.193	0.056	

Note

The same lower‐case letters within each column indicate that the difference between devices was statistically significant by Dunn‐Bonferroni.

N/A, Device not available in EU. Question not addressed in sufficient numbers in Japan.

Abbreviations: AECG, ambulatory ECG; D, Germany; UK, United Kingdom; USA, United States of America.

^†^ The comparisons among USA, EU, and Japan samples, Pearson's chi‐square test.

^¶^ According to the Bonferroni Correction a *P*‐value less than .0167 was considered statistically significant given three combinations and 0.008 for four comparison groups.

^‡^ The comparisons among types of technology within each cohort, Cochran's Q test.

### 3.3 Respondent assessment of AECG device attributes

**FIGURE 1:**
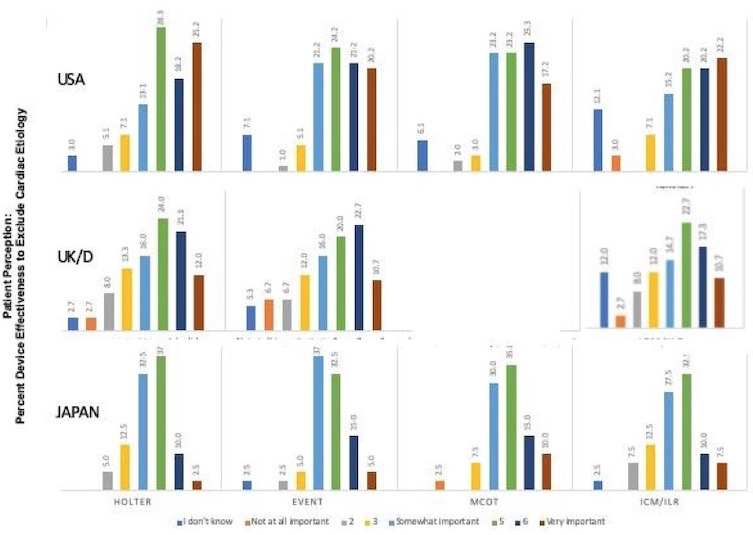
Bar graphs illustrating patient perceptions across the three geographies regarding the ability of specific AECG (Ambulatory ECG) technologies to identify the cardiac cause of syncope/collapse. The color‐coded columns represent patient's perception of importance (see definition of colors along the bottom). The ordinate is the percentage of patient responses for each column. MCOT (Mobile cardiac Outpatient Telemetry) was not available in UK/D (United Kingdom/Germany)

Respondents were queried with regard to selected attributes of the various diagnostic AECG technologies (Table 3). Findings reveal that patients did not identify major device attribute differences across technologies although there was a non‐significant trend for Holter monitors to rank higher than other devices for most attributes (Table 3). Thus, the ability to detect arrhythmic causes of syncope, convenience, and ease of us, and being discreet ranked comparably (Table 3). On the other hand, within technology categories, there were geographical differences. For instance, USA patients tended to be more confident that Holter monitors were effective for detecting an arrhythmia, and were more convenient to use than did UK/D or Japanese patients. Similarly, despite cost concerns discussed below, both USA and Japan patients gave ICM/ILRs higher scores than did UK/D patients (Table 3), but overall USA patients registered a more favorable view of ICM/ILRs for excluding an arrhythmic or cardiac cause of syncope, (T2B score 42.4%) compared to UK/D (T2B 28%) patients or Japan (T2B: 17.5%) patients (Figure 1).

**TABLE 3:** Patient perception of device attributes (Likert score, mean ± SD)

Perception (Likert rank) that device is effective for	n	Holter monitor	Event monitor	MCOT	ICM/ILR
Detecting if the cause is a heart rhythm disorder					
USA	99	5.2 ± 1.6^a^, ^b^	4.8 ± 1.8^a^	4.8 ± 1.8	4.7 ± 2.3^a^
UK/Germany (UK/D)	75	4.4 ± 1.8^a^	4.1 ± 1.9^a^	—	4.0 ± 2.1^a^
Japan	40	4.7 ± 1.1^b^	4.7 ± 1.1	4.6 ± 1.2	4.7 ± 1.3^a^
*P*‐value		**.003** ^†^	**.019** ^†^	.062^‡^	**.007** ^†^
Ability to rule out a cardiac cause					
USA	99	5.1 ± 1.7^b^	4.9 ± 1.8	4.9 ± 1.7	4.6 ± 2.2
UK/Germany (UK/D)	75	4.5 ± 1.7	4.3 ± 2.0	‐	4.0 ± 2.1
Japan	40	4.4 ± 1.1^b^	4.6 ± 1.3	4.8 ± 1.2	4.4 ± 1.5
*P*‐value		**.004** ^†^	.048^†^	.216^‡^	.063^†^
Not visible when worn					
USA	99	4.6 ± 1.8	4.3 ± 2.1	4.5 ± 2.0	4.6 ± 2.3
UK/Germany (UK/D)	75	4.1 ± 1.9	4.1 ± 1.9	—	4.0 ± 2.2
Japan	40	4.3 ± 1.2	4.4 ± 1.2	4.1 ± 1.3	4.5 ± 1.1
*P*‐value		.166^†^	.661^†^	.071^‡^	.063^†^
Convenient to use					
USA	99	5.1 ± 1.6^a^, ^b^	4.8 ± 1.8	4.8 ± 1.8	4.7 ± 2.1
UK/Germany (UK/D)	75	4.4 ± 1.7^a^	4.4 ± 1.9	—	4.1 ± 2.1
Japan	40	4.4 ± 1.4^b^	4.4 ± 1.3	4.5 ± 1.3	4.6 ± 1.2
*P*‐value		.002^†^	.079^†^	.055^‡^	.067^†^
Easy to use					
USA	99	5.1 ± 1.8^a^, ^b^	4.9 ± 1.8	4.9 ± 1.8	4.7 ± 2.1
UK/Germany (UK/D)	75	4.5 ± 1.7^a^	4.5 ± 1.7	—	4.1 ± 2.1
Japan	40	4.2 ± 1.3^b^	4.6 ± 1.2	4.4 ± 1.3	4.4 ± 1.3
*P*‐value		<.001^†^	.088^†^	**.005** ^‡^	.052^†^

^a^ USA and Japan vs UK/Germany (*P* < .05).

^b^ USA vs Japan (*P* < .05).

^†^ Kruskal‐Wallis test.

^‡^ Mann‐Whitney.

**FIGURE 2:**
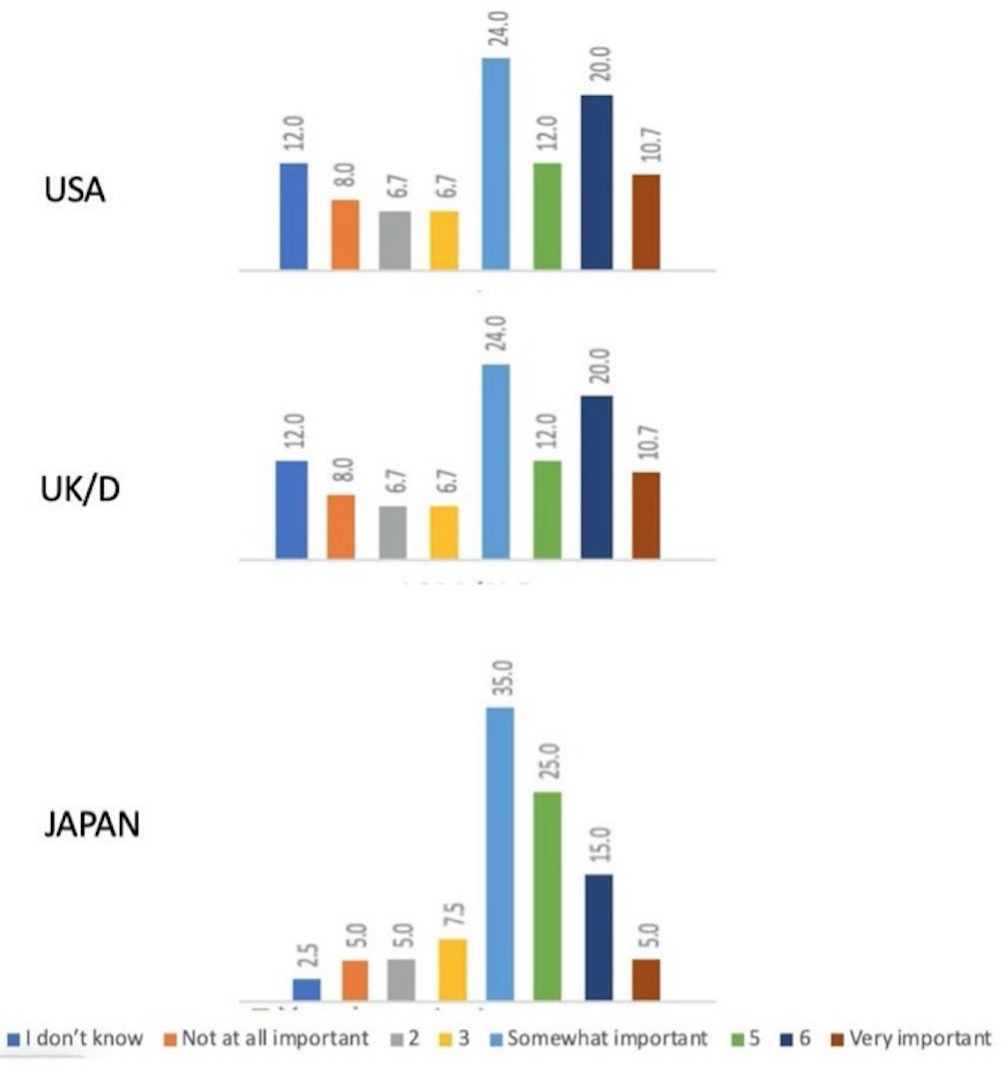
Bar graphs comparing across the three geographies patient concerns regarding ICM/ILR (Insertable cardiac monitors/loop recorders) cost. Likert scores are indicated in the color‐coded boxes under the graph. Based on T2B (Top 2 Box) scores, the cost was a greater concern in the USA and UK/D (United Kingdom/Germany) than in Japan

In regard to costs, both US and UK/D patients reported comparable concerns regarding ICM costs (T2B, 31%), and their concerns were greater than that of Japan patients (T2B score 20%) (Figure 2). The latter observations were surprising inasmuch as both Germany and Japan offer national health care schemes, while most of the US patients had yet to reach Medicare qualification age.

### 3.4 Patient education

The survey measured patient satisfaction with the information received from clinicians regarding prescribed AECGs (Table 4). Only those patients who had personally experienced a given technology were queried about that technology. Overall, patient rankings with respect to education did not differ significantly among devices. However, there was a trend for better scores for ICMs. The latter may have been due to the more invasive nature of the procedure and consequently the need to obtain informed consent. In addition, US patient rankings for the education they received regarding device choice and operation tended to be higher than UK/D and Japan patients, reaching statistical significance (*P* < .05) for Holters and ICMs when comparing the USA to Japan. Nonetheless, at their best, the Likert scores ranged only from approximately 4.7 to 6.0 across devices and geographies suggesting that there remained room for education improvement.

**TABLE 4:** Patient satisfaction about the education they received regarding various AECG devices

	n	Mean ± SD (Likert score)	*P*‐value^†^
Holter monitor			
USA	78	5.3 ± 1.5^a^	
UK/Germany	52	5.1 ± 1.4	NS
Japan	35	4.7 ± 0.8^a^	0.027
Event monitor			
USA	43	5.3 ± 1.3	
UK/Germany	37	4.8 ± 1.5	NS
Japan	21	4.9 ± 0.9	NS
ICM/ILR			
USA	30	6.0 ± 0.8^a^	
UK/Germany	19	5.2 ± 1.3	NS
Japan	10	4.9 ± 1.2^a^	0.015

Note

ICM/ILR: Insertable Cardiac Monitor/Implantable Loop Recorder.

^a^ USA vs Japan (*P* < .05).

^†^ Kruskal‐Wallis test.

## 4 DISCUSSION

This survey collected ‘real world’ data in an attempt to better understand patient perceptions of AECG monitoring technologies used for the evaluation of syncope/collapse. There were three main findings. First, the application of various AECG technologies was similar across the geographies, with a tendency for greater use of Holter monitors as part of the diagnostic process in the USA and Japan compared to the UK/Germany (UK/D). Second, findings reveal that patients did not identify major device attribute differences across technologies, although USA and Japanese patients tended to have a more favorable view of ICM/ILRs for excluding an arrhythmic or cardiac cause of syncope than did UK/D patients (Table 3). Finally, and possibly most importantly, patients across all geographies provided relatively low grades for the education that they received regarding the rationale behind clinician AECG recommendations. The latter low scores were most evident among Japan patients, but even in the USA and UK/D there was room for improvement. Since patient compliance is an important factor for AECG diagnostic success, the latter observation indicates that patients may benefit from more robust discussion by providers of the pros and cons of prescribed AECG technologies and the specific rationale behind the clinicians’ recommendations for a given individual's circumstance.

### 4.1 Factors determining AECG choice

The diagnostic effectiveness of available AECG technologies in the evaluation of syncope/collapse inherently depends on: (a) recording duration relative to the frequency of symptom recurrences as pointed out in both the USA and European practice guidelines,^3^, ^4^ (b) physician adherence to guideline recommendations, and (c) patient compliance. In regard to the first of these, multiple publications examining AECG use for arrhythmia diagnosis support the value of longer recording durations. For example, a number of studies document the greater diagnostic success achieved with extended ICM recordings in the evaluation of syncope or collapse compared to conventional ECM systems.^12^, ^13^, ^14^, ^15^, ^16^, ^17^, ^18^ However, in terms of physician adherence to guideline recommendations, there is some concern. For instance, Benditt et al^8^ examining USA experience, and Sutton et al^9^ assessing European data both found that while the majority of practitioners followed guideline recommendations, a substantial minority did not do so. A similar discrepancy was also reported by Sciaraffia et al based on a separate large European survey.^19^ On the other hand, apart from the brief experiential mention within the ISHNE‐HRS 2017 document,^20^ patient perceptions and understanding of AECG systems, and the impact that patient understanding might have on compliance with monitoring has received relatively little attention.

Smith et al^21^ examined patient preferences for a small centrally positioned patch electrode system when compared with a conventional Holter monitor electrode configuration. Patients noted less interference with the activity of daily living as well as less interference with sleep. They also preferred the discreet nature of the patch. Similarly, Sherr et al^22^ found that ‘leadless’ AECG systems proved effective with good compliance, but the patients were not subject to syncope/collapse in which the utility of such devices is limited.

The current study highlights the potential importance of patient education regarding the choice of cardiac monitoring systems for particular applications. Specifically, in the case of syncope/collapse, long‐term monitoring with recordings independent of patient interaction is important. Given these desirable requirements, physicians may lean toward MCOT systems^23^ and ICMs for diagnostic effectiveness. In this regard, and ignoring insurance limitations alluded to earlier, patients believe that they should receive more in‐depth careful explanation; it is expected that patient education will lead to greater acceptance of the inevitable inconveniences associated with AECG devices (eg, skin irritation, need to complete symptom diaries, invasive nature of ICMs).

### 4.2 Cost concerns

In this study, US and UK/D patients did register concern regarding ICM costs to a greater extent than did Japanese patients. While the reasons for cost issues were not addressed, US patient concerns may be due to the potential for more expensive technology to trigger greater out‐of‐pocket expenses since most patients were too young to qualify for government‐supported Medicare. The cost issues raised by UK/D patients are less easily accounted for as these patients are largely covered by national health care schemes; possibly, cost issues may reflect the possibility that more expensive technology might be denied by governing authorities. Further study of patient concerns regarding costs of AECG evaluation is warranted.

### 4.3 Limitations

Interpretation of the observations in this survey is subject to several important limitations. First, our findings are observational and therefore cannot be deemed to be as strong as those provided by randomized controlled trials. Nonetheless, the utility of ‘real world’ observations (ie, outside externally required constraints of clinical research trials) such as are presented here is receiving increased attention as has recently been highlighted by a multi‐society consensus report.^11^ Second, due to privacy constraints and the complexity of the survey, the patient sample in each geography was relatively small. Sampling magnitude was limited in part by the complexity of gathering data across diverse geographies; nonetheless, the sample size (see Methods) was deemed sufficient to offer a basis for designing future prospective studies. Third, responses were based on recollection of recent experience rather than prospectively accumulated. Inevitably, the order in which the various AECG technologies were used in a given individual will have impacted response. Fourth, patient co‐pay (ie, cost to the patient) varies from country to country and may have impacted patient perceptions of one technology versus another. Nevertheless, although ICMs would be expected to be the costliest to the patient, and did raise cost concerns (Figure 2), this possibility did not seem to exert a negative impact on patient opinions of ICM utility. Fifth, cost burden to patients may have affected which technology was used and some health insurers dictate which AECG technology is acceptable and in which order such technology may be applied. We did not address the latter two issues directly, but it is possible that patient experience may have been impacted by factors unrelated to device utility. Finally, patient responses may have been influenced by the respondents having been offered an honorarium. While the source of the honorarium was concealed, the respondent may have assumed that it was derived from an AECG manufacturer.

## 5 CONCLUSIONS

Findings across several geographies with quite different health insurance systems indicate that patients undergoing evaluation for syncope/collapse often lack understanding of differences among various AECG technologies. Providers are encouraged to offer more detailed patient education, focused on the rationale for AECG device selection. An informed patient is likely to exhibit greater investment in documenting his/her symptoms, and thereby be a more effective partner with the clinician in searching for a potential symptom‐arrhythmia correlation.

## CONFLICT OF INTEREST

Dr Benditt is a consultant to and holds equity in Medtronic Inc, and Abbott Laboratories, and is supported in part by a grant from the Dr Earl E Bakken family in support of Heart‐Brain research. Dr Sutton is a consultant to Medtronic Inc, serves on a Speaker's bureau for Abbott Laboratories (St Jude Medical, Inc), holds equity in Edwards LifeSciences Corp and Boston Scientific Inc. Dr Sakaguchi has been a consultant to BioTel Inc, an ambulatory ECG monitoring company. Robin Mears is an employee of Medtronic Inc, and holds equity in Medtronic Inc. Other authors have no conflicts to declare.

## AUTHOR CONTRIBUTIONS

Meltem Altinsoy: Preparation of manuscript, data analysis; Richard Sutton: Concept, revision of manuscript; Ritsuko Kohno: Data analysis, revision of manuscript; Scott Sakaguchi: Data review, manuscript revisions; Robin K. Mears: Collecting survey and technical data; David G. Benditt: Concept, manuscript preparation and revisions.
